# Supplementary material for: Psychopathic tendencies are selectively associated with reduced emotional awareness in the context of early adversity
Source: PLoS One. 2022 Dec 22;17(12):e0277475. doi: 10.1371/journal.pone.0277475 (PMC9778717; doi:10.1371/journal.pone.0277475)
Supplement: S1 File — (PDF) [file pone.0277475.s001.pdf]

Psychopathic tendencies are selectively associated with reduced emotional awareness in the context of early adversity

Ryan Smith, Anne E. Chuning, Colin A. Tidwell, John J.B. Allen, Richard D. Lane

Supplementary Materials

IRI Empathic Concern Analyses

In a Bayesian regression analysis assessing age, sex, CECA Total scores and LEAS Total scores (and their interactions) as possible predictors of IRI Empathic Concern scores, the most evidence was found for a model including sex (b = 1.47, CI = [0.73, 2.23), LEAS Total scores (b = 0.18, CI = [0.03, 0.34]) and an interaction between CECA and LEAS Total scores (b = 0.03, CI = [-0.0006, 0.07]): BF > 100 relative to an intercept-only model (extremely strong evidence). The 2nd-best model removed the interaction (BF = 0.47 relative to the winning model). Post-hoc analyses showed a positive relationship between LEAS Total scores and IRI Empathic Concern scores (r = .25, p < .001, BF = 34.4; see **Fig 1** in the main text), a nonsignificant relationship between CECA Total scores and IRI Empathic Concern scores (r = -.13, p = .10, BF = 0.65), and greater IRI Empathic Concern scores in females than males (t(171) = 4.92, 95% CI [2.2, 5.15], p < .001, d = .89, BF > 100). The interaction between LEAS and CECA scores was driven by a stronger positive relationship between LEAS scores and IRI Empathic Concern scores in those with higher CECA scores. This could be visualized by taking the same median split on CECA scores as in our previous analyses and correlating LEAS and IRI Empathic Concern scores for those with high vs. low CECA scores separately (see **Fig 2** in the main text). The correlation in

those with high CECA scores was *r* = .39 (*p* = .007, BF = 8.17), while the correlation in those with low CECA scores was *r* = .24 (*p* = .12, BF = .85).

To better understand the relationship between these measures, we subsequently conducted post-hoc correlations with CECA subscales (shown in **Fig 3** in the main text). These revealed that the negative relationship between IRI Empathic Concern and CECA Total scores was driven primarily by Mother Neglect scores.

In a Bayesian regression analysis assessing age, sex, CTQ Total scores and LEAS Total scores (and their interactions) as possible predictors of IRI Empathic Concern scores, the most evidence was found for a model including sex (b = 1.55, CI = [0.82, 2.30]), LEAS Total scores (b = 0.16, CI = [0.01, 0.32]), and an interaction between CTQ and LEAS Total scores (b = 0.05, CI = [-0.0005, 0.11]): BF > 100 relative to an intercept-only model (extremely strong evidence). The 2nd-best model removed the interaction (BF = .44 relative to the winning model). Subsequent analyses showed a nonsignificant relationship between CTQ Total scores and IRI Empathic Concern scores (*r* = -.13, *p* = .09, BF = .71). The interaction between LEAS and CTQ scores was driven by a stronger positive relationship between LEAS scores and IRI Empathic Concern scores in those with higher CTQ scores. This interaction was less apparent when taking the same median split on CTQ scores as in our previous analyses (See **Fig 2** in the main text). The correlation in those with high CTQ scores was *r* = -.40 (*p* = .005, BF = 11.85), while the correlation in those with low CTQ scores was *r* = -.30 (*p* = .12, BF = 1.20).

Post-hoc correlations (**Fig 3** in the main text) revealed that the positive relationship with LEAS Total scores was also present in both Self and Other subscales, and that there was a negative relationship between IRI Empathic Concern and CTQ physical abuse scores.

IRI Personal Distress Analyses

In a Bayesian regression analysis assessing age, sex, CECA Total scores and LEAS Total scores (and their interactions) as possible predictors of IRI Personal Distress scores, the most evidence was found for a model including LEAS Total scores (b = 0.27, CI = [0.09, 0.46]), an interaction between sex and CECA Total scores (b = 0.1 CI [-0.01, 0.22]), and an interaction between sex and LEAS Total scores (b = -0.25 CI = [-0.44, -0.06]): BF = 6.39 relative to an intercept-only model (moderate evidence). The 2nd-best model removed the interaction between sex and LEAS Total scores (BF = .75 relative to the winning model). A post-hoc Pearson correlation analysis showed a nonsignificant positive relationship between CECA Total scores and IRI Personal Distress scores (*r* = .12, *p* = .16; BF = .46). Post-hoc correlations with CECA subscales (shown in **Fig 3** in main text) suggested a weak positive relationship between IRI Personal Distress and CECA Father Antipathy scores. Because there was no interaction between LEAS Total scores and CECA Total scores, we did not examine separate groups of high vs. low CECA groups as we did with previous measures.

In a Bayesian regression analysis assessing age, sex, CTQ Total scores and LEAS Total scores (and their interactions) as possible predictors of IRI Personal Distress scores, the most evidence was found for a model including CTQ Total scores (b = 0.32, CI = [0.09, 0.55]), LEAS Total scores (b = 0.31, CI = [0.12, 0.50]), and an interaction between sex and LEAS Total scores (b = -0.24, CI = [-0.44, -0.05]): BF = 55.2 relative to an intercept-only model (very strong evidence). The 2nd-best model added a main effect of age (BF = .53 relative to the winning model). Post-hoc analyses showed a non-significant positive relationship between LEAS Total scores and IRI Personal Distress scores (*r* = .13, *p* = .10; BF = .67), a positive relationship between CTQ Total scores and IRI Personal Distress scores (*r* = .2, *p* = .01; BF = 4.49), and

nonsignificantly greater IRI Personal Distress scores in females than males ($t(171) = 1.68$, 95% CI [-0.22, 2.72], $p = .10$; BF = .68). The effects of sex and LEAS Total scores in the winning model were driven by their interaction, which showed a positive relationship in males ($r = .41$, $p = .009$; BF = 6.86), but no relationship in females ($r = .02$, $p = .83$; BF = .20; see **Fig 1** in the main text). To better understand the relationship between these measures, we subsequently conducted post-hoc correlations between the subscales for each of these measures (shown in **Fig 3** in the main text). These revealed that the magnitude of the relationship with LEAS Total scores was significant in both Self and Other subscales, and that the positive relationship between IRI Personal Distress and CTQ Total scores was driven primarily by emotional abuse. Because there was no interaction between LEAS Total scores and CTQ Total scores, we did not examine separate groups of high vs. low CTQ groups as with previous measures.

Based on the post-hoc median split analyses described in the main text, a two-sample t-test revealed that high-TPM/high-CECA individuals did not show significantly greater personal distress than high-TPM/low-CECA individuals (t(82) = 0.55, 95% CI [-1.39, 2.45], p = .59, d = .12, BF = 0.27). An analogous two-sample t-test revealed that high-TPM/high-CTQ individuals showed significantly greater personal distress than high-TPM/low-CTQ individuals (t(82) = 3.03, 95% CI [0.95, 4.6], p = .003, d = .69, BF = 11.12). The BF for the CTQ analysis provides strong evidence for the presence of this difference, but the BF for the CECA analysis provides poor evidence. This suggests there may be distinct subpopulations of high-psychopathy individuals that do or do not have a disposition to experience strong distress.

Low Empathy Analyses

As with analyses of high-psychopathy individuals in the main text, to selectively examine low-empathy participants we here performed a median split on IRI Empathic Concern scores (low <

22, high ≥ 22) and then restricted analyses to low-empathy participants. We then used the same Bayes factor model comparison approach as in our primary analyses. This allowed us to test for evidence of heterogeneity in EA within low-empathy individuals in relation to early adversity (i.e., consistent with primary vs. secondary psychopathy). Remaining high-empathy individuals were not the focus of these analyses (12/40 males, 79/133 females).

Using the CECA, the most evidence was found for a model including sex (b = 0.57, CI = [-0.01, 1.16]) and LEAS Total scores (b = 0.22, CI = [.08, .36]): BF = 99.28 relative to an intercept-only model (very strong evidence). The 2nd-best model removed the main effect of sex (BF = 0.30 relative to the winning model). Using the CTQ, the most evidence was found for a model including sex (b = 0.53, CI = [-0.07, 1.14]), LEAS Total scores (b = 0.21, CI = [0.06, 0.37]), and an interaction between CTQ Total scores and LEAS Total scores (b = -0.01, CI = [-0.05, 0.04]): BF > 100 relative to an intercept-only model (extremely strong evidence). The 2nd-best model removed the interaction (BF = 0.65 relative to the winning model).

For further interpretation and visualization of these interactions (see **Fig 4** in the main text), the high-psychopathy individuals were then divided into groups with low vs. high levels of early adversity, which allowed additional examination of EA in those with profiles more consistent with primary vs. secondary psychopathy. To do so, we performed a median split on CTQ (low < 6, high ≥ 6) and CECA (low < 14.75, high ≥ 14.75) Total scores. We then divided participants into two groups for each measure: low-empathy/high-early adversity (CTQ: 16/39 males, 35/132 females; CECA: 16/39 males, 30/133 females) and low-empathy/low-early adversity (CTQ: 11/39 males, 18/132 females; CECA: 11/39 males, 24/133 females).

As can be seen in **Fig 4** of the main text, those with low empathy and high early adversity showed positive relationships between IRI Empathic Concern scores and LEAS Total Scores

(low-empathy/high-CECA: *r* = .45, *p* = .002; BF = 28.31; low-empathy/high-CTQ: *r* = .31, *p* = .03; BF = 2.7). Those with low IRI Empathic Concern scores and low early adversity also showed positive relationships between IRI Empathic Concern scores and LEAS Total Scores, although this was not significant for the CECA (low-empathy/low-CECA: *r* = .24, *p* = .17; BF = .84; low-empathy/low-CTQ: *r* = .51, *p* = .005; BF = 11.05). For the interested reader, we note that remaining participants with high empathy scores also showed no relationship between IRI Empathic Concern scores and LEAS Total Scores (*r* = -.01, *p* = .90; BF = .24).

A two-sample t-test further revealed that low-empathy/high-CECA individuals showed significantly greater negative affect on the PANAS than low-empathy/low-CECA individuals (*t*(78) = 2.95, 95% CI [1.55, 8.00], *p* = .004, *d* = .66, BF = 9.08). An analogous two-sample t-test revealed that low-empathy/high-CTQ individuals showed significantly greater negative affect on the PANAS than low-empathy/low-CTQ individuals (*t*(78) = 2.88, 95% CI [1.48, 8.16], *p* = .005, *d* = .67, BF = 7.71). The BFs for these analyses provided moderate evidence for the presence of these differences.

A similar pattern of results was found for IRI Personal Distress scores. Namely, a two-sample t-test revealed that low-Empathy/high-CTQ individuals showed significantly greater distress than low-Empathy/low-CTQ individuals (*t*(78) = 2.49, 95% CI [0.46, 4.09], *p* = .015, *d* = .58, BF = 3.32). In contrast, an analogous two-sample t-test found a nonsignificant difference in distress between low-Empathy/high-CECA individuals and low-Empathy/low-CECA individuals (*t*(79) = 0.91, 95% CI [-1.71, 1.91], *p* = .91, *d* = .03, BF = .23). The BFs for these analyses provide moderate evidence for the presence of the difference between low- and high-CTQ groups, and evidence against the difference between low- vs. high-CECA groups. These results

suggest there may be distinct subpopulations of low-empathy individuals that do or do not have a disposition to experience strong distress.

In contrast, a two-sample t-test did not reveal differences in LEAS Total scores between low-empathy/high-CECA individuals and low-empathy/low-CECA individuals ($t(79) = -.55$, 95% CI [-2.3, 1.30], $p = .58$, $d = .12$, BF = .27). Similarly, a two-sample t-test also did not reveal differences in LEAS Total scores between low-empathy/high-CTQ individuals and low-empathy/low-CTQ individuals ($t(78) = .41$, 95% CI [-1.49, 2.24], $p = .69$, $d = .09$, BF = .27). It is notable that these BFs provided evidence for the absence of an effect, as opposed to merely a null result.

Post-hoc comparisons of other outcome measures between low-empathy/high-early adversity and low-empathy/low-early adversity individuals are shown in **Table S1**. Notably, unlike Meanness and Disinhibition, TPM Boldness was higher in those with Low CECA/CTQ scores; PANAS Positive Affect was higher in those with low CECA scores; Empathic Concern was higher in those with low CECA scores; and LEAS Self scores were higher in those with low CECA/CTQ scores. Although not shown in **Table S1**, comparison of low- and high-empathy individuals did not find that low-empathy individuals had experienced greater early adversity overall with respect to CTQ Total scores (t(169) = -1.74, 95% CI [-1.49, 0.09], p = .08, d = 0.27, BF = 0.67) or CECA Total scores (t(170) = -1.34, 95% CI [-2.56, 0.44], p = .16, d = 0.21, BF = 0.41).

Table S1. Summary statistics (mean and SD) of measures in low-Empathy group by early adversity level.

**Measures**[a]	**High CECA (N = 46)**	**Low CECA (N = 35)**	p^b	**Cohen's *d***	**Bayes Factor**
**TPM Total**	72.52 (18.18)	61.91 (15.88)	**0.007**	**0.61**	**5.66**
**TPM Boldness**	30.65 (7.52)	32.37 (8.73)	0.345	0.21	0.34

**TPM Meanness**	17.63 (8.81)	12.89 (6.98)	**0.011**	**0.59**	**4.30**
**TPM Disinhibition**	21.39 (9.99)	14.60 (7.68)	**0.001**	**0.75**	**24.43**
**IRI Empathic Concern**	17.35 (2.95)	17.97 (2.88)	0.334	0.22	0.35
**IRI Personal Distress**	12.50 (4.31)	12.40 (3.67)	0.913	0.03	0.23
**PANAS Negative Affect**	24.58 (7.76)	19.480 (6.37)	**0.004**	**0.66**	**9.08**
**PANAS Positive Affect**	33.67 (7.46)	34.97 (6.433)	0.187	0.41	0.32
**Age**	19.24 (1.90)	19.03 (1.34)	0.578	0.13	0.27
**LEAS Total**	32.59 (4.39)	33.09 (3.50)	0.583	0.12	0.27
**LEAS Self**	27.57 (5.52)	29.40 (3.76)	0.095	0.79	0.79
**LEAS Other**	26.61 (4.70)	26.37 (4.67)	0.822	0.05	0.24
**Measures**	**High CTQ (N = 51)**	**Low CTQ (N = 29)**	***p***	**Cohen's *d***	**Bayes Factor**
**TPM Total**	69.49 (18.28)	65.69 (17.43)	0.366	0.21	0.34
**TPM Boldness**	29.90 (7.72)	33.72 (8.17)	**0.04**	**0.49**	**1.52**
**TPM Meanness**	16.67 (8.72)	14.17 (7.23)	0.196	0.30	0.50
**TPM Disinhibition**	20.20 (10.14)	15.59 (8.13)	**0.04**	**0.49**	**1.55**
**IRI Empathic Concern**	17.78 (2.70)	17.34 (3.20)	0.515	0.15	0.29
**IRI Personal Distress**	13.27 (4.02)	11.00 (3.74)	**0.015**	**0.58**	**3.32**
**PANAS Negative Affect**	24.24 (7.845)	19.41 (6.76)	**0.005**	**0.67**	**7.71**
**PANAS Positive Affect**	33.88 (7.17)	34.86 (6.69)	0.549	0.14	0.28
**Age**	19.18 (1.87)	19.14 (1.30)	0.922	0.02	0.24
**LEAS Total**	33.00 (4.29)	32.62 (3.50)	0.687	0.09	0.26
**LEAS Self**	28.49 (4.93)	28.24 (4.98)	0.829	0.05	0.25
**LEAS Other**	26.84 (4.92)	25.97 (4.26)	0.424	0.19	0.32

[a]Data from the TPM, IRI, and PANAS are novel to this study. Data from LEAS and CTQ have previously been described [1].
[b]p-values are based on two-sample t-tests between those with high vs. low CTQ or CECA scores (based on median splits).
[c]Note that, after quality control checks, the final N regarding high vs. low CECA analyses changed for the PANAS (N = 90).

Proportion of Individuals with High Psychopathy and High Emotional Awareness

To assess the proportion of individuals with high psychopathy and high emotional awareness, we took a median split on LEAS Total scores and a median split on TPM Total scores across the sample. Combining these median splits showed that there were 41 individuals (23.3% of the full sample) with scores greater than or equal to the median score for both LEAS Total scores and TPM Total scores (9/40 male, 32/136 female). This indicated that 46.6% of individuals with

psychopathic traits also had high EA, and that 42.5% of individuals with high EA also had psychopathic traits.

Proportion of Individuals with Low Empathy and High Emotional Awareness

To assess the proportion of individuals with low empathy and high emotional awareness, we also took a median split on LEAS Total scores and a median split on IRI Empathic Concern scores across the sample. Combining these median splits showed that there were 41 individuals (23.2% of the full sample) with scores greater than or equal to the median score for LEAS Total scores and lower than the median for IRI Empathic Concern scores (10/40 male, 31/136 female). This indicated that 50% of individuals with low empathy also had high EA, and that 42.3% of individuals with high EA also had low empathy.

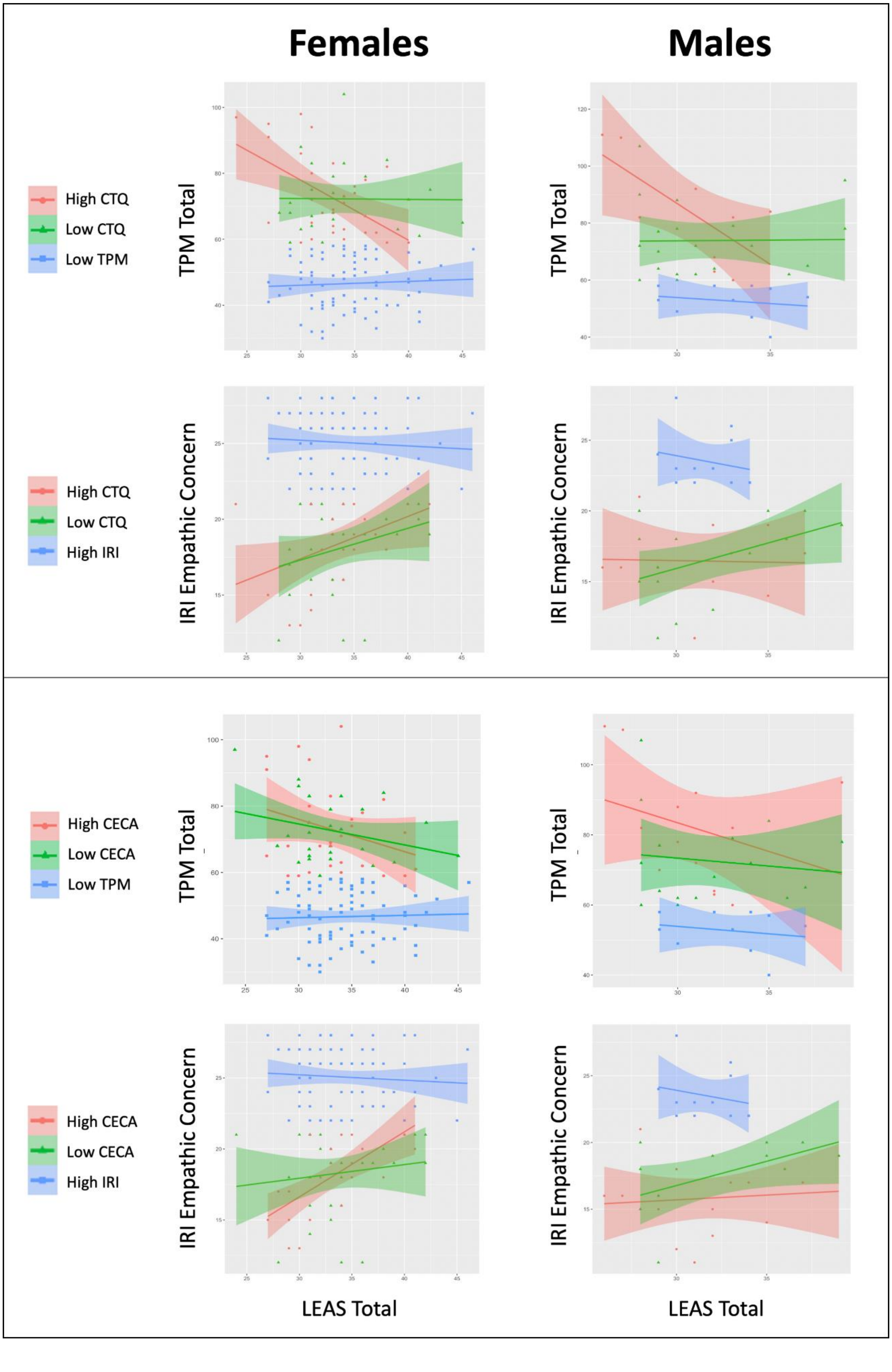
Females
Males
High CTQ
Low CTQ
Low TPM
TPM Total
High CTQ
Low CTQ
High IRI
IRI Empathic Concern
High CECA
Low CECA
Low TPM
TPM Total
High CECA
Low CECA
High IRI
IRI Empathic Concern
LEAS Total
LEAS Total

S1 Fig. **Relationships between emotional awareness, psychopathy/empathy, and early adversity when separated by sex.** This figure is analogous to **Fig 4** in the main text but with results separated by sex. It shows correlations in high-psychopathy/low-empathy groups between Levels of Emotional Awareness (LEAS) Total Scores and both Triarchic Psychopathy Measure (TPM) Total scores and Interpersonal Reactivity Index (IRI) Empathic Concern scores, separated by high vs. low levels of early adversity (based on median splits). Early adversity measures included the Childhood Experiences of Care and Abuse questionnaire (CECA) and the Childhood Trauma Questionnaire (CTQ). Unlike in **Fig 2** in the main text, high and low early adversity levels were taken only from individuals with scores above and below the median for the TPM and IRI, respectively. The blue lines illustrate the correlations in the remaining low psychopathy and high-empathy individuals. As can be seen, the pattern of results was similar in males and females.

References

1. Smith R, Steklis HD, Steklis N, Weihs K, Allen JJB, Lane RD. Lower emotional awareness is associated with greater early adversity and faster life history strategy. Evolutionary Behavioral Sciences. 2022:ebs0000282. doi: https://doi.org/10.1037/ebs0000282.
