# Supplementary material for: Psychopathic tendencies are selectively associated with reduced emotional awareness in the context of early adversity
Source: PLoS One. 2022 Dec 22;17(12):e0277475. doi: 10.1371/journal.pone.0277475 (PMC9778717; doi:10.1371/journal.pone.0277475)
Supplement: Attachment — Submitted filename: Responses.docx [file pone.0277475.s003.docx]

**Responses to reviewers**

Editor Comments:

I too have had the opportunity to review the manuscript, and am in broad agreement with the reviewer regarding the relative strengths and weaknesses of the manuscript. I believe the reviewer comments to be both clear and well-regarded, and so will not repeat them here. I will however expand somewhat on their concern regarding the method used to separate primary versus secondary psychopathy in the study. Whereas the reviewer has requested that you provide additional justification for these methods, I would request that you go one (or two) steps further than this, and also consider (regardless of your ability to find historical support for the method) its appropriateness in the present context. I do understand the logic – in fact, as someone who has recently been working with the PD-scale from the IRI, I quite like the logic of relating it to primary/secondary psychopathy concepts. However, relating it to primary/secondary psychopathy, and *defining it as* primary/secondary psychopathy are not the same thing.

**Reply #1: Thank you, these are very helpful points. We agree that it needs to be clearer that personal distress scale score is not a rigorous means of identifying individuals with primary vs. secondary psychopathy. To help address this issue, we have added/edited language to stress this point. We have removed any language suggesting that secondary psychopathy is defined as having high negative affect scores or personal distress (although we have referenced work [e.g., see ref 76] that discusses secondary psychopathy as being primarily differentiated in terms of high anxiety due to early adversity). We also agree with the reviewer that the personal distress scale is not a straightforward indicator of secondary psychopathy (its correlations with our other measures suggest it is most related to a disposition toward high-anxiety and negative affect, with little-to-no relation to empathic concern [r=.14, p=.06, BF=.99]). We have now framed their theoretical motivation more appropriately. And we now examine personal distress mainly in the context of supplemental analyses for the interested reader. In general, the paper now focuses more on how the relationship between emotional awareness and psychopathy/empathic concern is dependent on early adversity. These are more cautiously referred to as being consistent with (but not definitive of) primary vs. secondary psychopathy. Here are a couple examples of updated wording:**

**Pg. 6 - *“In this study we addressed the potential relationship between EA and psychopathy and hypothesized that an association would be present. However, based on the work reviewed above, we expected that this relationship could differ between those with vs. without early adversity (i.e., associated with secondary vs. primary psychopathy profiles, respectively; e.g., see (46)). Based on the common link to early learning, we hypothesized that EA would be negatively correlated with psychopathy scores in those with early adversity (i.e., consistent with secondary psychopathy). Given the mixed literature regarding emotional abilities in primary psychopathy, we predicted the presence of a relationship in those with high psychopathy scores without early adversity, but did not have a strong directional hypothesis.”***

**Pg. 22 - *“This suggested that the initially observed negative relationship between EA and psychopathy scores was driven primarily by those with early trauma and negative affect (i.e., more consistent with a secondary psychopathy profile).”***

Moreover, in the present context, which is a study that relies on the reporting of relationships between different self-report measures, I wonder if your use of the PD scale in this manner doesn’t add substantive bias into the study design. You are, after all, predicting that psychopathy will be related to reduced emotional awareness, and are then using a measure of personal distress – which is established as a substantive correlate of emotional awareness – to demonstrate that only a subset of your psychopathy group shows this reduced EA. It all fits, it all makes sense…but is it in fact a somewhat circular process, wherein the category being used to separate participant groups is related to the dependent measure being evaluated for differences between groups (see Kriegerskorte et al., 2008)?

**Reply #2: This point about potential circularity is well-taken. It is true that some measures of emotional awareness (e.g., alexithymia using the self-reported TAS-20) are reliably correlated with distress and negative affect. This is one major reason we instead focus on the levels of emotional awareness scale (LEAS), which is often used in psychosomatic medicine research specifically because it is not confounded by personal distress and negative affect (i.e., while it does show associations with several medical/psychiatric conditions). This has been shown in several studies. Most importantly here, the LEAS is also not correlated with personal distress scores in our sample. As shown in Fig 3, LEAS shows a non-significant positive correlation (r=.12) with personal distress. So, if anything, it would bias results in the opposite direction. We think it is important to emphasize this point more, as it is a very natural concern to have. To do so, we have added/revised language to make this clearer. However, as described in Reply #1, we have also de-emphasized/re-framed some analyses using the PD scale that were the point of concern. Here are examples of how we’ve stressed the independence of LEAS and personal distress, and how we’ve tried to clarify the role of the PD scale in the analyses we have kept:**

**Pg. 14 - *“Notably, LEAS scores were not significantly related to IRI personal distress scores, consistent with prior studies indicating that, unlike alexithymia measures (e.g., (74)), LEAS is independent of negative affect (75). Similarly, LEAS Total scores were also not related to PANAS negative affect scores (r = .09, p = .24, BF = .34) or positive affect scores (r = .1, p = .2, BF = .38).”***

**Pg. 22 - *“In further support of the existence of heterogeneity within psychopathic profiles in our sample, we found that those with high psychopathy scores and early adversity reported greater negative affect than those without early adversity. In contrast, these two groups did not show significant differences in EA. As observed previously, LEAS scores were also independent of negative affect and distress (75).”***

**Pg. 16 - *“For the interested reader, we provide detailed results of analyses (analogous to those above) for IRI personal distress within Supplementary Materials. There was a notable interaction between sex and LEAS Total scores in predicting IRI Personal Distress scores, which showed a positive relationship in males (r = .41, p = .009, BF = 6.86), but no relationship in females (r = .01, p = .92, BF = .20; see Fig 1). These analyses were considered exploratory, as we did not have strong a priori hypotheses regarding this measure. However, given the observed pattern of relationships between this measure and CTQ emotional abuse, TPM Boldness, and PANAS scores (see Fig 3), it might be viewed in this context as a convergent measure of dispositional negative affect (i.e.,*** ***more consistent with the high-anxiety states associated with secondary psychopathy (76)).”***

In addition, while I agree with the reviewer that your description of Bayes Factors was clear and helpful, I am nonetheless left wondering if those BF analyses were necessary. Perhaps some additional language that does more than simply explain how to interpret the BFs, but also informs on how they help further interpretation beyond the reported r and t-statistics (assuming they do) would be helpful.

**Reply #3: We agree it could be helpful to make sure the benefits of the statistical approach are clear. One important benefit is interpretability. When only computing p-values within a frequentist approach, all one can do is either reject or fail to reject the null hypothesis. In contrast, the Bayesian approach evaluates the probability of the data under a null model as well as other models. This means you can calculate evidence *for* a null model as opposed to simply failing to reject it (e.g., a BF = 1/3 indicates 3 times more evidence for a null model than for an alternative model, while BF = 1 indicates equal evidence for null and alternative hypotheses). This is useful in the present case, since it indicates not only where tested relationships are likely present but also where they are likely absent. Another benefit is model comparison. By identifying the model with the most evidence, this provides a way of selecting which predictors should be included in subsequent analyses. We have added/revised some language as follows to clarify this:**

**Pg. 10 - *“A BF represents the ratio of the probability of observed data under one model vs. another (i.e., where a higher probability of data under a model provides more evidence for that model). For example, BF = 1/3 indicates that data are three times more likely under the null model than the alternative model, BF = 1 indicates equal evidence for the null and alternative models, and BF = 3 indicates that the data are three times more probable under the alternative model than the null model. When interpreting the strength of evidence of each result, we adopt the guidelines described in Lee and Wagenmakers (71): BF = 1-3, poor/anecdotal evidence for the alternative hypothesis; 3-10, moderate evidence; 10-30, strong evidence; 30-100, very strong evidence; >100, extremely strong evidence. In cases of extremely strong evidence, we will indicate BF > 100 (as these values can get cumbersomely large under default priors in large sample sizes or when one model fits much better than another); we further compare BFs for the 1st- and 2nd-best model to offer a better sense of the relative evidence of the most competitive models. These BF analyses provide several advantages. Most importantly here, they provide a straightforward basis for model selection (i.e., indicating which predictors are relevant to include), and they allow the evaluation of evidence for the null model as well as for alterative models. Thus, they improve interpretability by allowing us to gain confidence that a relationship between variables is absent vs. present (i.e., as opposed to simply failing to reject a null hypothesis).”***

**We also make several statements in the manuscript indicating when our results provided evidence against the presence of specific relationships, which we think strengthens the contribution of the analyses.**

Review Comments to the Author

Reviewer #1: This manuscript reports results from a study examining the relationship between psychopathic traits (as measured by TriPM) and emotional awareness (EA; as measured by LEAS), including examination of “primary” and “secondary” psychopathy in relation to EA. The authors report their findings of apparent EA deficits in psychopathy, particularly in a subgroup of participants with high TriPM scores who reported experiencing early adversity (as measured by CECA and CTQ). The questions about empathy, EA, and psychopathy addressed by the research are interesting and important to furthering understanding of the relationship between those variables. Additionally, the authors did a good job of describing the Bayes factor analysis statistical methodology, providing sufficient information for readers less familiar with the BF method to understand the reported results. It was helpful to have access to the supplementary information provided by the authors, and I commend the authors on their clear delineation between a priori and post-hoc analyses. Despite the many strengths of this manuscript, there are some areas that warrant revisions, particularly adding support for certain methodological decisions and improving some aspects of the interpretation of the study results. Should the authors revise the document to reflect the feedback below, the manuscript would likely be suitable for publication and constitute a valuable addition to the published literature in this area.

Major Concerns

(1) Questionable definitional/methodological choices for assessing constructs of interest

A. The chosen way of defining and measuring “primary” vs. “secondary” psychopathy needs additional explanation/empirical support. Have other studies delineated the two subtypes of psychopathy in this way? Please include citations that support the chosen way of defining and measuring these constructs (e.g., Explain why using the CECA, CTQ, PANAS, and IRI personal distress subscale to distinguish between psychopathic subtypes is a valid method.).

**Reply #4: We agree with the reviewer that it was not appropriate to define primary/secondary psychopathy based on early adversity and negative affect measures. Indeed, many papers evaluate primary vs. secondary psychopathy based on additional psychopathy features (although, high-anxiety in response to early adversity is often discussed as a central characteristic for distinguishing secondary psychopathy; e.g., see ref 76 in the manuscript). As such, we have revised language to make it clear that early adversity and negative affect are only features consistent with primary vs. secondary psychopathy, and are not definitive. We provided a few quotes of updated language in Reply #1 and #2 to exemplify these revisions.**

i. The use of the IRI personal distress subscale as a “convergent measure of primary vs. secondary psychopathy” (p. 9) was odd and requires additional explanation/justification. As the personal distress subscale of the IRI is one of the affective/emotional empathy subscales, the authors will need to add to their justification and explanation for the hypothesis that this affective empathy subscale would be associated with the “secondary” form of psychopathy.

**Reply #5: As describe in Replies #1 and #2, we agree that interpreting the personal distress subscale as a convergent measure of secondary psychopathy is not straightforward. We have therefore removed this framing/discussion of this subscale. It is now primarily used to provide secondary insights for the interested reader in supplementary materials. However, we also added some language indicating – based on its correlations with other measures and past characterizations in previous studies – why it might be seen as reflecting dispositions toward the high-anxiety states associated with secondary psychopathy. But we make no claims that personal distress is a strong indicator of secondary psychopathy.**

**Pg. 16 - *“…given the observed pattern of relationships between this measure and CTQ emotional abuse, TPM Boldness, and PANAS scores (see Fig 3), it might be viewed in this context as a convergent measure of dispositional negative affect (i.e., more consistent with the high-anxiety states associated with secondary psychopathy (76)).”***

**Note that this section references Fig 3, which shows that distress is higher with emotional abuse, lower with TPM Boldness, higher with greater negative affect, and lower with greater positive affect. This makes sense if you look at the specific items of the scale, which do not reflect concern for others, but simply the tendency to enter high-anxiety states in situations where harm is present.**

B. In the analyses looking at psychopathy subtypes, the authors included high psychopathy/high early adversity, high psychopathy/low-early adversity, and low psychopathy groups.

i. The choice to use median splits on the various measures to create the subgroups for analyses needs to be explained and supported with citations, including an explanation on how this choice may have impacted the results.

ii. The choice to not compare high and low early adversity in the “low psychopathy” group must be explained, including any impact this choice may have had on the results and interpretations of the findings. Further, the authors should include results of analyses showing whether the high and low psychopathy groups differed with regard to levels of experienced early adversity.

**Reply #6: We thank the reviewer for highlighting these helpful points. One thing that should be better emphasized/explained in the manuscript is that most median split analyses were done largely for secondary interpretive/visualization purposes. Our primary results assessed the interaction between LEAS and early adversity (CTQ/CECA) in predicting psychopathy scores. The significant interaction found in our models indicated a stronger negative relationship between LEAS and psychopathy scores in those with greater early adversity. However, as these are all continuous measures, this interaction is hard to illustrate. Our solution was to show correlations using the median splits as a way to visualize how the relationship between EA and psychopathy differed in the high vs. low early adversity groups. Most analyses performed on the median split groups were therefore secondary. We are not aware of specific citations to provide for previous work that has used median splits on the TPM for similar purposes.**

**In some other secondary analyses, we used a median split to selectively examine individuals with high psychopathy scores. These were also done largely for purposes of interpreting our primary results, by** **confirming whether the relationship to EA still depended on early adversity when only looking at those high in psychopathic traits. This is the case the reviewer refers to when stating that we divided individuals into 3 groups (i.e., low psychopathy, and high psychopathy with vs. without early adversity). However, it is more appropriate to say that we selectively examined the two high-psychopathy groups, and that the low-psychopathy participants were not the focus of these analyses. In other words, we were trying to answer a question about heterogeneity within those high in psychopathic traits, so the low-psychopathy participants were not relevant to this question.**

**To make this clearer, we have adjusted language in several places to make sure it is clear that: (1) median splits were used either simply for visualization or for interpretation of our primary results, and (2) low-psychopathy participants were not relevant to questions about heterogeneity within psychopathic populations, and were therefore not the focus of those analyses. We also added in the requested analyses of early adversity differences in high vs. low psychopathy groups (and for low vs. high empathy groups in supplementary materials). We also acknowledged potential limitations of the choice to use median splits in the discussion. Here are a couple examples of relevant language in the revised manuscript:**

**Pg. 13 - *“The interaction between LEAS and CECA scores was driven by a stronger negative relationship between LEAS scores and TPM scores in those with higher CECA scores. This could be visualized by taking a median split on CECA scores (low < 14.75, high ≥ 14.75) and correlating LEAS and TPM scores for those with high vs. low CECA scores separately (see Fig 2). Although displayed here primarily for purposes of visualization, we note that the correlation in those with high CECA scores was r = -.42 (p < .001, BF > 100), while the correlation in those with low CECA scores was r = -.12 (p = .29, BF = .42). These correlations were also significantly different (z = -2.14, p = .03).”***

**Pg. 17 - *“To more thoroughly interpret the significant interactions between psychopathy, early adversity, and emotional awareness identified in our primary analyses above, we performed additional post-hoc analyses to selectively probe these relationships in participants with high psychopathy scores. To do so, we performed a median split on TPM scores (low < 59, high ≥ 59) and then restricted analyses to high-TPM participants. We then used the same analysis approach as above to test if the interaction between LEAS scores and early adversity scores was still present in predicting psychopathy scores. This allowed us to test for evidence of heterogeneity in EA within high-psychopathy individuals in relation to early adversity (i.e., consistent with primary vs. secondary psychopathy). Note that our question here was specifically about the possibility of distinct patterns in individuals showing high psychopathy scores; thus, the low-psychopathy individuals (below the median for TPM scores; 10/40 males, 78/137 females) were not included in these analyses.***

***As in our main analyses above, the winning models still included this interaction…***

***As above, for further interpretation and visualization of these interactions (see Fig 4), the high-psychopathy individuals were then divided into groups with low vs. high levels of early adversity, which allowed additional examination of EA in those with profiles more consistent with primary vs. secondary psychopathy. To do so, we performed a median split on CTQ (low < 6.67, high ≥ 6.67) and CECA (low < 16.5, high ≥ 16.5) Total scores. We then divided participants into two high-TPM groups for each measure: high-TPM/high-early adversity (CTQ: 10/40 males, 33/137 females; CECA: 14/40 males, 31/137 females) and high-TPM/low-early adversity (CTQ: 20/40 males, 26/137 females; CECA: 16/40 males, 28/137 females).***

***Illustrating the significant interactions described above, Fig 4 shows that those with high psychopathy and high early adversity showed significant negative relationships between TPM Total scores and LEAS Total Scores…”***

**Pg. 18 - *“Comparison of high- and low-psychopathy individuals also confirmed that high-psychopathy individuals had experienced greater early adversity overall with respect to CECA Total scores (t(175) = 3.73, p < .001, d = 0.56, BF = 88.73) and CTQ Total scores (t(175) = 2.93, p = .004, d = 0.44, BF = 8.3).”***

**Pg. 24 - *“This also relates to limitations in our selective analyses of high-psychopathy individuals based on a median split. Namely, while those scoring above the median reflect higher levels within a student population, this may not capture high levels in a community or prison population. Other ways of dividing individuals into high and low groups were also possible.”***

(2) Interpretation of study results and explanation in the Discussion could be stronger

A. The authors used the TriPM as their psychopathy measure, and I would have liked to see more discussion of the subscale-level results, as it appears that the results would be in-line with the authors’ hypotheses about intact EA associated with “successful psychopaths” or those adept in manipulation. Specifically, findings that the negative relationship between LEAS Total Score and TriPM psychopathy was driven by the Meanness and Disinhibition subscales (and not Boldness; p. 13), suggests that individuals high in Boldness (but lower on the other two subscales), might have intact EA. The discussion would benefit from more explanation of the TriPM subscale findings, particularly as they relate the authors’ hypotheses about “primary” and “secondary” psychopathy.

i. Including the TriPM subscales in the conceptualization of “primary” vs. “secondary” psychopathy in the introduction could help tie this together.

ii. This could be brought up in the Discussion as well. For example, the authors stated, “The weaker association with EA in primary psychopathy could help account for these individuals’ prowess at displaying superficial charm, manipulating others, and attaining leadership roles, while the stronger association in secondary psychopathy is consistent with the high impulsivity and high criminality seen in this population.” The authors would be able to make a stronger statement if they mentioned the potential role of Boldness scores in this distinction. Table 2 (p. 18) shows that the two “high psychopathy” groups differed at the subscale level, so highlighting this would make the discussion of the findings stronger.

**Reply #7: This is a very good point, which we had not thoroughly considered. We have now added discussion of this, as suggested. For example:**

**Pg. 22 - *“Another interesting result of this study was that LEAS scores were associated with the meanness and disinhibition subscales of the TPM, but not with the boldness subscale. The meanness and disinhibition subscales largely reflect features associated with secondary psychopathy, such as aggression, impulsivity, irresponsibility, and oppositionality. In contrast, the boldness subscale reflects features more consistent with primary psychopathy, such as dominance, persuasiveness, social assurance, resiliency, and intrepidness. The selective lack of relationship between LEAS and boldness could therefore be seen as further evidence supporting the idea that EA is not associated with a primary psychopathy profile.*** ***In addition, high-psychopathy individuals with high early adversity showed lower boldness than those with low early adversity, which also supports the presence of distinct psychopathy profiles within the sample.”***

B. There are a few places where non-significant results (with p > .05) are still interpreted, for example being described as “marginal” effects (e.g., p. 16). This type of questionable practice takes away from the manuscript as a whole, and is unnecessary, so use of such interpretations of the results in the manuscript should be reconsidered.

**Reply #8: We appreciate this point. In all cases, we have now removed statements about marginal results and simply state that these results are non-significant.**

C. The discussion of the findings for “primary” vs. “secondary” psychopathy (p. 20) was confusing. Specifically, the authors mention that LEAS scores for individuals in the high psychopathy/high early adversity group did not significantly differ from those in the high psychopathy/low early adversity group, but then go on to discuss how their results show support for a difference in EA between the two groups. A clearer explanation of these findings is warranted.

**Reply #9: We apologize for the lack of clarity. In the revised manuscript, we have revised wording to make the relevant points clearer. The important point is that only the early adversity group shows a strong relationship between psychopathy scores and emotional awareness. So the groups don’t show differences in mean levels of EA. Instead, the group difference we are discussing is meant to highlight that the level of psychopathy is only related to EA in the high early adversity participants. Throughout the discussion, we have taken care to always say the difference is in these relationships, and not that there is a group difference in mean levels of EA. Here is an example revised paragraph:**

**Pg. 22 - *“In further support of the existence of heterogeneity within psychopathic profiles in our sample, we found that those with high psychopathy scores and early adversity reported greater negative affect than those without early adversity. In contrast, these two groups did not show significant differences in EA. As observed previously, LEAS scores were also independent of negative affect and distress (75). Roughly one third of individuals with high psychopathy scores also showed levels of EA above the median value (and analyses using a separate measure also showed that over one third of low-empathy individuals showed EA levels above the median value; see Supplementary Materials). Therefore, high psychopathy (and low empathy) did not prevent individuals from displaying high EA in general. However, further analyses in high-psychopathy individuals revealed that EA and psychopathy were strongly negatively correlated in those with high early adversity, whereas high-psychopathy individuals without early adversity showed little-to-no relationship between these variables. A somewhat similar pattern of results was also seen when analyzing a second measure of empathy levels. This suggested that the initially observed negative relationship between EA and psychopathy scores was driven primarily by those with early trauma and negative affect (i.e., more consistent with a secondary psychopathy profile).”***

Minor Concerns

(1) In the very first sentence of the introduction (p. 3), it says “Psychopathy is a personality trait characterized by...”. First, it would be more accurate to refer to psychopathy as a “constellation of traits” rather than just a singular trait (though that may have been a typing error in the manuscript). The definition of psychopathy as being “characterized by antisocial tendencies, such as cruelty, manipulation, grandiosity, callousness, and lack of empathy” (p. 3) is a bit questionable, particularly since you used the TriPM, which has reduced emphasis on the “antisociality/criminality” component of psychopathy (unlike the PCL-R).

**Reply #10: We have now revised the wording to refer to a “constellation of antisocial personality traits”, along the lines of the reviewers’ suggestion. Otherwise, our revised opening description of psychopathic traits simply aims to list the range of dispositions associated with the general construct of psychopathy. In our view, this is appropriate as a broad introductory description, where the focus then becomes more specific as the paper progresses. If the editor feels this introductory description requires further editing, we are happy to consider this in a subsequent revision. The revised version states:**

**Pg. 3 - *“Psychopathy refers to a constellation of antisocial personality traits, such as cruelty, manipulativeness, grandiosity, callousness, and lack of empathy (1). Individuals with psychopathy are also characterized as being high in egocentrism/narcissism, exploitativeness, impulsivity, aggression, and criminality, while showing low remorse and fear, and shallow affect more generally (2, 3).”***

(2) The mention of and emphasis on “dark triad” traits in the introduction (e.g., p. 3) was surprising and a bit confusing, seeing as the abstract and title focus on psychopathy. It seems that the arguments made in the introduction to set up the rationale behind the authors’ own study could have been made through sticking with the psychopathy literature. If the inclusion of “dark triad” traits is essential to the study, adding more explanation of the different constructs and how the psychopathy literature may differ from the “dark triad” literature would be helpful.

**Reply #11: We agree that explicit description of the dark triad concept may have distracted from the main focus. The dark triad mainly divides the broader set of traits associated with psychopathy into three constructs that also include Machiavellianism and Narcissism. We felt this literature should not be ignored in our review (as it includes many studies of psychopathic traits), but also agree that it may not be helpful to discuss the separate triad constructs in detail. To ameliorate this, we have removed explicit discussion of the dark triad and kept the focus on the broader psychopathy construct. However, we still cite some of the dark triad literature when it offers relevant results in relation to psychopathy more broadly.**

(3) The authors appear to use an “emotional empathy” definition of “empathy” throughout the manuscript; however, the explanation of this definition is lacking. Specifically, on page 4, it says “Despite deficits in empathy (or “affective theory of mind”; i.e., emotion contagion in response to others’ emotions) ...”. The terms of “emotional contagion,” “affective theory of mind,” and “empathic concern” all seem to be used interchangeably in this manuscript; however, the constructs are not necessarily considered equivalent in the literature. I would suggest clarifying your definitions of empathy by clearly laying out your definitions of both “cognitive empathy” and “affective empathy,” as you use both in the manuscript and study design/results interpretation.

**Reply #13: We appreciate this suggestion. It is true that the literature does not always use these terms consistently. Some papers we cite use “cognitive empathy” in a way that is synonymous with cognitive theory of mind (as applied to others’ emotions). Others use “affective theory of mind” in a manner similar to “emotional” empathy (i.e., caring that another person is sad and being motivated to make them feel better – where in some sense it “hurts” to see others sad, which relates to a type of emotion contagion). Our choice of terms was driven mainly by consideration of how these terms were used in the papers we cited. But we agree it could be helpful to revise the wording in hopes of making definitions a bit clearer. As such, we have revised the section as follows:**

**Pg. 3 - *“One common question pertains to the relationship between psychopathy and socio-emotional abilities. On the one hand, empathy deficits in psychopathy (i.e., lack of concern for others) might be expected to hinder such abilities. On the other hand, the manipulation skills, superficial charm, and successful attainment of leadership roles in psychopathy each suggest a proficiency in detecting and capitalizing on the emotions of others (7, 9, 11, 12). Individuals with psychopathic traits have also been purported to use empathy as a manipulative strategy (13-15). However, the literature on this topic is mixed. For example, while psychopathy is associated with a reduced tendency to feel negative emotion in response to the suffering of others (i.e., affective empathy; sometimes also called affective theory of mind), some studies in those with psychopathic traits have observed intact abilities to recognize the thoughts or feelings of others (i.e., cognitive theory of mind or cognitive empathy; e.g., see (16-18)). Yet, other studies find that psychopathic traits are negatively correlated with these cognitive abilities (13, 14, 19-22). Studies examining emotional intelligence (EI) also find mixed results, with intact EI present in some studies (and/or assessment measures) but not others (e.g., (23-25)).”***

(4) The authors cite study time constraints and their a priori hypotheses (p. 9) as the reason for not including the two cognitive empathy subscales from the IRI (Perspective Taking, Fantasy); however, this choice seems to require more explanation. Specifically, the authors made efforts to include measures for convergent validity for the psychopathy subtyping, so it was odd that they would leave the 14 items from the two cognitive empathy subscales of the IRI out. It seems like it would have been helpful to include these subscales, and the justification for not including these items seemed insufficient, particularly because the authors highlight this as a study limitation (p. 21).

**Reply #14: We agree that including the cognitive subscales may have offered some interesting secondary insights. But these were not directly related to the hypothesis being tested. Our main hypothesis was about the relationship between EA and psychopathy (using TPM scores). As a secondary analyses, we were interested in a convergent measure of the affective empathy deficit in psychopathy, due to its particular relevance to our question about EA. The IRI empathic accuracy subscale seemed like a good tool for this purpose, and we collected personal distress as well for largely exploratory purposes. If we had more time, we would have liked to include the cognitive subscales, but time constraints required compromise here. Since our main hypothesis was not about cognitive theory of mind abilities in psychopathy, we judged these scales were the least problematic to remove to remain within our study time limits. It was mentioned as a limitation simply because it could have helped address other secondary questions of interest. But not because it necessarily limited how we could interpret our results themselves. With this in mind, we have made some revisions to language in a couple places in hopes of making it clear that they could offer secondary insights but were not central to our stated hypothesis. For example:**

**Pg. 9 - *“The IRI (67) assesses both cognitive and affective components of dispositional empathy (2 subscales each). Based on our a priori hypotheses, and the study’s time constraints, we only assessed the two affective subscales: Empathic Concern (having sympathy for others in need) and Personal Distress (having negative arousal in response to perceived distress in others). While the two cognitive subscales had the potential to provide secondary insights, they were not prioritized because they were not required to assess the hypothesized relationship between EA, early adversity, and psychopathy/low affective empathy.”***

**Pg. 23-24 - *“These considerations highlight some important limitations of the current study and point toward future directions. First, we did not collect measures of emotion recognition or cognitive theory of mind, which prevented us from assessing whether previously observed differences in these related abilities were also present in our sample. While not related to our primary hypotheses, such measures could have offered additional insights. It would be helpful for future studies to replicate our findings while also including such measures, so that potential inconsistencies with previous work can be addressed.”***

(5) There appears to be a typing error on page 19, in the “Low Empathy Analyses” section, where it mentions “IRI Empathic Accuracy scores,” and I believe it is supposed to say “IRI Empathic Concern scores.”

**Reply #15: Thanks for pointing out this typo. It has now been fixed.**

Editorial office comments:

1. Please ensure that your manuscript meets PLOS ONE's style requirements, including those for file naming. The PLOS ONE style templates can be found at

https://journals.plos.org/plosone/s/file?id=wjVg/PLOSOne_formatting_sample_main_body.pdf and

https://journals.plos.org/plosone/s/file?id=ba62/PLOSOne_formatting_sample_title_authors_affiliations.pdf.

**Reply #16: We have now ensured that manuscript adheres to the style requirements.**

2. Thank you for stating the following in the Funding Section of your manuscript:

“R.S. is supported by the William K. Warren Foundation and the National Institute of General Medical Sciences (P20GM121312).”

We note that you have provided funding information that is not currently declared in your Funding Statement. However, funding information should not appear in the Acknowledgments section or other areas of your manuscript. We will only publish funding information present in the Funding Statement section of the online submission form.

Please remove any funding-related text from the manuscript and let us know how you would like to update your Funding Statement. Currently, your Funding Statement reads as follows:

“R.S. is supported by the William K. Warren Foundation (https://www.williamkwarrenfoundation.org/) and the National Institute of General Medical Sciences (P20GM121312; https://www.nigms.nih.gov/). The funders had no role in study design, data collection and analysis, decision to publish, or preparation of the manuscript.”

Please include your amended statements within your cover letter; we will change the online submission form on your behalf.

**Reply #17: We have removed the funding statement from the manuscript and now provided these details in the cover letter, as requested.**

3. We noted in your submission details that a portion of your manuscript may have been presented or published elsewhere. [In a separate paper under review, we also use the data on emotional awareness and early adversity to answer a separate research question about how early adversity influences emotional awareness. We do not reproduce any of those analyses here and explicitly acknowledge this other paper in our manuscript. In this manuscript we instead examine how emotional awareness measures relate to psychopathy measures and empathy measures, and whether early adversity and negative affect moderate those relationships. The data on psychopathy, empathy, and negative affect has not been used in any previous study. A preprint of this other paper can be found here: https://psyarxiv.com/7nzqk/] Please clarify whether this [conference proceeding or publication] was peer-reviewed and formally published. If this work was previously peer-reviewed and published, in the cover letter please provide the reason that this work does not constitute dual publication and should be included in the current manuscript.

**Reply #18: This paper has now been published. The citation has now been included in the manuscript. It does not constitute dual publication for the reasons mentioned above. Namely, we use several measures not used in the previous paper, and all analyses/results reported in this paper are novel and were not included in the previous paper. We have now explained this in the cover letter.**

4. We note that you have referenced (Patrick CJ. Operationalizing the triarchic conceptualization of psychopathy: Preliminary description of brief scales for assessment of boldness, meanness, and disinhibition. Unpublished test manual Tallahassee, FL,: Florida State University; 2010.which has currently not yet been accepted for publication. Please remove this from your References and amend this to state in the body of your manuscript: as detailed online in our guide for authors

http://journals.plos.org/plosone/s/submission-guidelines#loc-reference-style ".

**Reply #19: We have now replaced this citation with a published paper covering the same topic.**
