# Supplementary material for: Psychopathic tendencies are selectively associated with reduced emotional awareness in the context of early adversity
Source: PLoS One. 2022 Dec 22;17(12):e0277475. doi: 10.1371/journal.pone.0277475 (PMC9778717; doi:10.1371/journal.pone.0277475)
Supplement: Attachment — Submitted filename: Response to Reviewers.docx [file pone.0277475.s004.docx]

**We’d like the thank the editor and reviewers for these helpful additional comments. We’ve indicated each of our responses in BOLD below.**

Reviewer #1: This manuscript reports results from a study examining the relationship between psychopathic traits (as measured by TriPM) and emotional awareness (EA; as measured by LEAS), including examination of “primary” and “secondary” psychopathy profiles as they may relate to EA. The authors report their findings of apparent EA deficits in psychopathy, particularly in a subgroup of participants with high TriPM scores who reported experiencing early adversity (as measured by CECA and CTQ). The questions about empathy, EA, and psychopathy addressed by the research are interesting and important to furthering understanding of the relationship between those variables. The authors did a great job of addressing the concerns raised during the initial review through edits to the manuscript. Aside from some minor concerns, outlined below, I believe the manuscript is suitable for publication and will constitute a valuable addition to the published literature in this area.

Minor Concerns:

1) In the description of the various measures used (starting on page 7), I noticed that Cronbach’s alpha is included for the LEAS measure, but not for the other measures. The authors might consider adding Cronbach’s alpha values for the subscales of the TriPM, and the other measures, as possible and appropriate.

**Reply: We appreciate the reviewer’s suggestion and agree it would be useful to provide Cronbach’s alpha values for each measure. Consistent with what was we preciously included for the LEAS, we have now also provided information on the internal reliability for the TPM, CECA, CTQ, and PANAS as was reported in the initial validation studies.**

2) There appears to be a typing error on page 21, in the “Secondary analyses in low-empathy participants” section, where it mentions “IRI Empathic Accuracy scores,” and I believe it is supposed to say “IRI Empathic Concern scores.”

**Reply: We thank the reviewer for noticing this typo. It has now been corrected.**

3) On page 22, in the Discussion section, the following sentence presented a bit of confusion: “In contrast, these two groups did not show significant differences in EA.”
(a) When reading this sentence, I interpreted the two groups to be the high-TPM/high-early adversity and high-TPM/low-early adversity based on the preceding sentence that mentions heterogenous psychopathic profiles in the sample. If this is a proper interpretation, the sentence expressing that there was no significant difference in EA between the groups is confusing given other statements that seem to suggest that the two groups did differ in EA, with the high-TPM/high-early adversity group having lower EA (lower LEAS scores) and the high-TPM/low-early adversity group not showing the same “impairment” in EA. For example, in the abstract, the final sentence reads: “This suggests that EA may be selectively associated with levels of secondary psychopathy, while those with high levels of primary psychopathy remain capable of high EA.” This sentence in the abstract seems at odds with the idea that there was no difference in EA between the groups being referenced in the above-mentioned sentence on page 22.
(b) It is possible my interpretation of this sentence or the results is incorrect, though I think some clarification would be helpful to avoid similar confusion in others who will read this.

**Reply: We appreciate this concern and agree that it was unclear. We decided to remove this sentence from the manuscript to avoid potential confusion for future readers.**

4) On page 23 of the Discussion section, the following sentence is included: “The selective lack of relationship between LEAS and boldness could therefore be seen as further evidence supporting the idea that EA is not associated with a primary psychopathy profile.”
(a) The authors could clarify this sentence by specifying that “diminished EA” (or “impaired EA” or some other synonym) does not seem to be associated with a primary psychopathy profile.
(b) For example, the sentence could be worded like this: “The selective lack of relationship between LEAS and boldness could therefore be seen as further evidence supporting the idea that diminished EA is not associated with a primary psychopathy profile.”

**Reply: We appreciate the reviewer’s suggestion and agree that their proposed wording would help increase the clarity of the sentence. We have now revised it as suggested (p. 25):**

*“The selective lack of relationship between LEAS and boldness could therefore be seen as further evidence supporting the idea that diminished EA is not associated with a primary psychopathy profile.”*

5) Additionally, on page 23 of the Discussion section, the following is included and seemed a bit confusing: “However, it appears less consistent with work showing that individuals with primary psychopathy, but not secondary psychopathy, show emotion recognition deficits [15, 46] or with studies showing preserved “cognitive” theory of mind abilities for emotions of others [16-18].”
(a) The point of confusion is related to the second part of the above sentence. Specifically, it is unclear to me how the results of the present study are not consistent with studies showing preserved cognitive empathy/cognitive theory of mind in primary psychopathy.
(b) I would suggest removing this part of the sentence or clarifying how the results are inconsistent with those referenced previous study findings on cognitive empathy in primary psychopathy.

**Reply: As suggested, we have now deleted this sentence from the manuscript.**

Reviewer #2: The authors address an interesting issue. The question of how adverse early experiences may contribute to adult psychopathology is an important one. The paper is generally well-written. However, along with these strengths, there are some weaknesses. For example, the sample is relatively small and the methodology is a somewhat roundabout way to address the question – using childhood adversity and lower empathy to signal secondary psychopathy rather than addressing the sub-facets of psychopathy directly. In what follows, page numbers refer to the clean document from the reviewing PDF. I do not object, in principle, to the authors analyzing data from the same participants who contributed to a different publication on a different hypothesis, but milking data from a sample of 177 participants into two papers does feel a bit exploitative especially when the three measures included in the previous publication were analyzed again here.

**Reply: We appreciate the reviewer’s feedback on the manuscript. With respect to our prior paper, we would like to clarify that, while we understand the concern, all analyses in the present paper were novel and the hypothesis explored in this manuscript was a primary motivator in study design and data collection. Several of the main measures (i.e., the TPM, IRI, and PANAS) were not examined in our prior paper and are thus entirely novel to the present report. In our view, not examining these variables in the dataset would have been wasteful and a missed opportunity. While our previous report examined the LEAS and the early adversity measures, this was done to address a very different question and thus no analyses in that previous report were replicated here. We have ensured that this is stated explicitly in the manuscript (p. 12):**

*“We note here that data on LEAS, CTQ, and CECA scores have been analyzed in a previous study [64]. The data shown from TPM, IRI, and PANAS scores, and their relations to EA and early adversity, are novel to this study.”*

Just because there is an association whereby those with lower LEAS scores had higher TPM and lower IRI scores, does not suggest that those with lower TPM and higher IRI scores had HIGH LEAS scores per se – Associations indicate how relatively higher or lower scores covary but do not indicate whether scores are high relative to average scores, for example. So, the authors might think about rephrasing the last sentence of the abstract. It should also be clear throughout that the sample is not a clinical sample.

**Reply: We appreciate these suggestions and agree that it is important to present these aspects of the methods/results as clearly as possible. We have ensured that it is clearly stated in the manuscript that it is a student sample and not a clinical sample. The last sentence of the abstract has also been edited to better represent the findings. It now reads as follows:**

*“This suggests that lower EA may be selectively associated with higher levels of secondary psychopathy, while those with higher levels of primary psychopathy remain capable of higher EA.”*

On p. 4, the authors contrast primary and secondary psychopathy by referring to insufficient arousal to emotional cues as being emblematic of primary psychopathy and referring to secondary psychopathy as stemming from early adversity. This is a contrast of inequivalences of sorts. The authors should focus on either early etiology or associations with emotion awareness rather than comparing apples to oranges.

**Reply: We agree with the reviewer’s feedback on the wording of the sentence in question and appreciate the opportunity to make the full comparison more explicit. The sentence (p. 4) has now been edited and reads as follows:**

*“One well-established distinction is between primary and secondary psychopathy – the former being associated with insufficient arousal to emotional cues, but no specific relationship to early adversity or negative affect, while the latter is associated with hyperarousal to emotional cues and elevated negative affect, and thought to develop in response to early abuse and neglect [29-39].”*

We can’t know if secondary psychopaths are incarcerated at higher rates without knowing the prevalence of both types both in the general population and in prison populations. All we can know is whether a higher rate of tested prisoners are characterized as secondary rather than primary psychopaths. The authors still need to be more precise in their language. For example, on p. 5 (end of first paragraph), they mention the etiology of psychopathic traits. Do we know that adverse experiences contribute to the development of such tendencies, or do they just covary? Authors have to be careful when making causal assumptions based on correlations. It is possible that, if there is a genetic component, parents high in psychopathy are both more likely to abuse their children and pass on psychopathic traits, meaning that maltreatment itself is not an etiological factor. All of these ideas are interesting and important, but require a great deal of care in how they are described.

**Reply: We agree it is important to be clear about this. In response to the reviewer’s specific concern about criminality and incarceration rates, we have now edited the relevant sentence to reflect the findings more appropriately (p. 4):**

*“Primary psychopathy is characterized by callous, selfish, deceitful, ruthless, and manipulative behaviors (i.e., associated with “successful” psychopaths), while secondary psychopathy is characterized by risk-taking behavior, impulsivity, short-term thinking, antisocial behavior, and violent criminal behavior (and are more frequently found in prison populations than individuals with primary psychopathy).”*

**Regarding the reviewer’s concern that adverse experiences may co-vary with secondary psychopathy in the absence of a causal relationship, we decided to edit the relevant text in a couple ways. First, we more explicitly highlighted that the cited studies were longitudinal (i.e., Dunedin cohort and FACHS study) and more strongly support causal interpretations than simple cross-sectional correlations. At the same time, we acknowledged that non-causal interpretations remain possible. The relevant text (p. 4-5) now reads as follows:**

*“For example, epigenetic modifications to oxytocin receptor systems associated with prosocial behaviors have been observed specifically in children with primary psychopathy profiles [41], while longitudinal studies suggest that learning maladaptive emotional behaviors in adverse childhood environments may play an important role in the development of secondary psychopathy [42, 43].* *Here it is important to highlight, however, that other non-causal interpretations remain possible (e.g., if psychopathic traits inherited from parents simultaneously promote childhood maltreatment), and the nature of these relationships could also differ in those with distinct psychopathy profiles.”*

P. 5 at reference [44], less accurate compared to what? The authors should specify when making comparisons.

**Reply: We have now changed the wording in this sentence to improve clarity. The sample in question compared a sample of maltreated children to peers without reported maltreatment. The sentence (p. 5) now reads as follows:**

*“For example, maltreated children appear to show greater responses to emotional faces (consistent with secondary psychopathy), but are less accurate at labelling them in comparison to peers without reported maltreatment [44].”*

P. 5 – there should be citations following the statement that trait EA is widely recognized…

**Reply: As requested, the sentence (p. 5) now includes citations to three supporting references:**

*“Trait EA is widely recognized as an important individual difference variable in mental health research [47-49].”*

The hypothesis at the bottom of p. 5 (that there are different reasons to suspect both high or low emotional awareness in individuals with psychopathy and other dark traits) has been suggested by others (e.g., Vonk et al., 2013 with regard to narcissism, Esperger & Bereczkei, 2012; Nagler et al., 2014; Vonk et al., 2015).

**Reply: We appreciate the reviewer’s recommendation to include additional references here. After reviewing the two suggested articles that were not already included in the manuscript, we agree that they are appropriate to include. An excerpt of this addition (p. 6) reads as follows:**

*“Others have also hypothesized that primary and secondary psychopathy differ in their relationships with specific socioemotional skills, such that those with primary psychopathy are suggested to be more skilled at emotional manipulation, emotional control, and spontaneous mentalization, as well as to possess higher emotional intelligence [19, 22, 67, 68].”*

It might be helpful to frame in terms of the distinction between ability to read emotion states (cognitive empathy) and the capacity to feel for others (emotional or affective empathy). It is not clear to me whether EA is capturing the latter or a form of emotional intelligence and how this should relate to theory of mind and empathy more broadly. Because much of the prior literature is couched in terms of this distinction, it would help for these authors to clarify the relations among these various related constructs. For example, they mention using the subscales of the IRI as a convergent measure of empathy but is LEAS really a measure of empathy? I am not personally familiar with the LEAS. Suddenly, the PANAS is mentioned as a measure of affect in the Method section but it is unclear why each construct is included.

**Reply: We appreciate the need to keep these distinctions clear. There are some additional challenges posed by the fact that different areas of the literature use the terms “emotion recognition”, “cognitive empathy”, “cognitive theory of mind”, “affective empathy”, and “affective theory of mind” (among others) in partially overlapping ways. Our aim throughout the manuscript has been to succinctly highlight these overlapping constructs and stick to a consistent choice of terminology throughout. We agree that the constructs being measured in this study could be more explicitly linked to measures described within the methods sections, which has led us to make edits toward that goal. To answer the reviewer’s questions, the LEAS is best understood as a measure related to emotional intelligence. For example, as described in the updated scale description in the methods, and in the introduction, it relates to fine-grained conceptualization of emotional responses in self and others with links to emotion recognition and “affective theory of mind” (i.e., theory of mind for the emotions of others). That said, in the absence of psychopathic tendencies, one would expect greater empathic concern (“affective empathy”) in those that are more aware of the emotions of others. To clarify, however, the IRI empathic concern scale was seen primarily as a convergent measure of psychopathy, due to the fact that low affective empathy is a central feature of psychopathy. The PANAS Negative Affect scale was included to assess the elevated negative affect expected in those with secondary psychopathy, as reviewed in the introduction. As one example of the edits we have made to improve clarity here, the associated section of text (p. 8) now reads as follows:**

*“As a second measure of the low empathy associated with psychopathy, we also collected data from affective empathy-related scales in the Interpersonal Reactivity Index (IRI; [72]). Additionally, the Positive and Negative Affect Schedule (PANAS-20; [73]) was included to measure expected differences in negative affect associated with primary vs. secondary psychopathy… The 10-item LEAS [50, 51] is a widely used performance measure of EA. It can be understood to measure the cognitive granularity/complexity with which individuals conceptualize/understand the emotions of self and others, which is often considered an important aspect of emotional intelligence… Other-related scores can be understood to relate to aspects of cognitive empathy or the overlapping construct of theory of mind for emotions. Crucially, while high scores for this subscale reflect greater granularity in how an individual conceptualizes the emotions of others, it does not necessarily indicate greater affective empathy (e.g., a person could be aware that another individual is in distress without experiencing negative emotions in response).”*

It would help to see a formalization of the conceptual model proposed by the authors before the Method. The authors should also spend some time indicating how the construct of psychopathy is captured by the TPM compared to other measures that have been used in previous studies. Do the authors care about the overall TPM score or the subscales? Do the subscales represent primary and secondary psychopathy? If not, how do they fit into the conceptual model for the study?

**Reply: We appreciate these suggestions. We believe the clarifications and other statements provided within the introduction, in part based on our responses to several other comments here, should help clarify the model we have in mind. Specifically, we expect primary psychopathic traits (i.e., high TPM scores [esp. the Boldness subscale] combined with lack of negative affect or early adversity) to be less associated with emotional awareness, while secondary psychopathic traits (i.e., high TPM scores [esp. the meanness and impulsivity subscales] combined with elevated negative affect and early adversity) should be related to emotional awareness, because early adversity may both hinder development of emotional awareness and promote psychopathic traits in some individuals. We believe this model is articulated clearly in the introduction of the revised manuscript (especially in the final two paragraphs). An explicit example statement in the revised manuscript, for example, the last two paragraphs of the introduction (p. 6-7) state:**

*“…early adversity is negatively related to EA [64], and EA is thought to reflect adaptive early learning [48, 65], both of which could suggest a negative relationship between EA and secondary psychopathy; that is, early adversity could both hinder development of EA and contribute to secondary psychopathy… In this study we addressed the potential relationship between EA and psychopathy and hypothesized that an association would be present. However, based on the work reviewed above, we expected that this relationship could differ between those with vs. without early adversity (i.e., associated with secondary vs. primary psychopathy profiles, respectively; e.g., see [46]). Based on the common link to early learning, we hypothesized that EA would be negatively correlated with psychopathy scores in those with early adversity (i.e., consistent with secondary psychopathy)…”*

**We have also addressed the reviewer’s request to describe the TPM scale in greater detail and provide readers with an overview of self-report psychopathy measurement. We have also included clarifying language to demonstrate the subscales’ relevance to primary and secondary psychopathy as well as our ultimate decision to use the total score for initial analyses. For example, within the methods section, we have added the following paragraph (p. 9):**

*“The TPM was selected to measure psychopathy within this study because it is based on the contemporary triarchic model of psychopathy, an effort to integrate differing concepts of psychopathy proposed by Patrick and colleagues [74].Other self-report measures of psychopathy, such as the Psychopathic Personality Inventory (PPI; [75]), the Levenson Self-Report Psychopathy Scale (LSRP; [34]), and the Antisocial Process Screening Device (APSD; [76]), vary in their conceptualization of psychopathy and its traits, leading to potential inconsistencies when comparing results across different studies. While we appreciate these tools, their specific origins, and this existing body of work, we chose the TPM here because it offers a balanced measure of multiple distinct dimensions of psychopathy. For a more in-depth discussion and review of the differences between leading self-report psychopathy measures, the interested reader may see the following: [77-80]. Additionally, in line with our hypothesis about differences in primary and secondary psychopathy, the TPM includes two subscales related to these two presentations. Higher boldness is typically associated with primary psychopathy, while higher impulsiveness is typically associated with secondary psychopathy [81]. However, we were also interested in the relationship between EA and overall levels of psychopathic tendencies. Therefore, initial analyses focused on total scores; further analyses then examined subscale scores to better understand the specific dimensions of psychopathy that best explained initially observed patterns of relationships between variables.”*

Near the bottom of p. 9, is psychopathy/low affective empathy meant to treat these two as synonymous? It is difficult to evaluate the statistical approach without greater clarity from the authors on the model.

**Reply: We appreciate the reviewer’s request for clarity on the sentence in question. Our intention with the forward slash between psychopathy and low affective empathy was to distinguish these from the other constructs being listed. As mentioned above, we assume low affective empathy is one core feature of psychopathy. We have now edited this sentence to make it clearer. The sentence (p. 11) now reads as follows:**

*“While the two cognitive subscales had the potential to provide secondary insights, they were not prioritized because they were not required to assess the hypothesized relationship between EA, early adversity, and psychopathy, as well as the low levels of affective empathy associated with psychopathy more broadly.”*

In addition, the measures of childhood adversity appear to focus on a somewhat narrow range of experiences related to abuse rather than more broadly capturing things like poverty, exposure to dangerous environments, illness etc.

**Reply: We agree that early adversity can be conceptualized in broader and narrower terms. The CTQ and the CECA are widely-used measures in the field of early adversity research, and it is often assumed that abuse and neglect are central to this construct. The methods section now provides greater justification for their use. However, we agree with the reviewer that this conceptualization of early adversity is not comprehensive. Therefore, we have provided further description of these limitations within the discussion section.**

**The addition (p. 10) to the methods section reads as follows:**

*“The CECA and the CTQ were selected to measure early adversity, as both follow the conceptual framework of the initial Adverse Childhood Experiences (ACES) study [82]. The CTQ has good convergent validity with the ACES measure and research has shown that it is more sensitive at capturing different types of early adversity [83].”*

**The addition (p. 27) to the discussion section reads as follows:**

*“Additionally, our measurement of early adversity, while standard within existing literature, does not measure some other early environmental factors that could impact development, such as illness, poverty, environmental toxins, etc. Future research should explore these other aspects as they relate to psychopathy. Life history strategy is another construct which could be explored within the context of psychopathy and emotional awareness, as previous literature shows connections to both [64, 104].”*

If the scoring for the LEAS came from a computer scoring method, where does the inter-rater reliability come from?

**Reply: This is a good point. The inter-rater reliability reported in the methods section was referring to the original hand scoring version of the LEAS. This language has now been removed, as these data were scored with the computer scoring method.**

The authors should report reliability for all measures.

**Reply: We agree and have now provided information on the internal reliability for the TPM, CECA, CTQ, and PANAS as was reported in the initial validation studies.**

P. 9, why do the authors say “the empathic concern subscale acted as a convergent measure of low affective empathy in addition to the TPM”? The TPM doesn’t measure empathy???!!

**Reply: In response to the reviewer’s concern, we have edited this sentence to represent our thought process more clearly. In fact, the meanness subscale of the TPM has items that overlap considerably with the construct of affective empathy (with higher meanness corresponding to lower empathy). The sentence (p. 12) now reads as follows:**

*“The Empathic Concern subscale acted as a convergent measure of the low affective empathy associated with psychopathy; the content of this subscale overlaps with the Meanness dimension measured in the TPM.”*

I don’t find the rationale for the personal distress scale to be compelling as described on p. 9-10.

**Reply: We were unsure as to exactly why the reviewer found this to not be compelling, and so found it difficult to confidently address this comment in detail. The rationale proposed within the methods section explains that the inclusion of the IRI Personal Distress subscale was exploratory and primarily included in supplementary analyses for the interested reader. Additionally, we reference our use of the PANAS, which should lead readers to recall that elevated negative affect is expected to be seen in those with secondary but not primary psychopathic traits, and the items of the Personal Distress subscale have content that overlaps considerably with negative affect and elevated responsiveness to emotional cues.**

Confidence intervals should be reported for t-tests.

**Reply: At the reviewer’s request, we have added confidence intervals for all t-tests reported in the main text as well as in the supplementary materials.**

How was the sample size determined? Is the student sample even representative and valid for the questions posed? The authors address this issue in the discussion but it is a serious limitation of the research.

**Reply: We agree that the sample characteristics lead to limitations and have made efforts to ensure that this is highlighted appropriately. In response to this comment, we have added additional information in the methods section to describe the sample size determination. We have also expanded the discussion of the student sample’s limitations within the discussion section, which included reviewing and referencing past literature using the TPM in different populations. We have also amended the abstract to say “undergraduate students” instead of “individuals” when describing the sample.**

**The description of the participant sample in the methods section (p. 7) now reads as follows:**

*“A convenience sample of 177 students (40 male) at the University of Arizona (mean age = 19.07, SD = 1.82 years), was recruited from Tucson, AZ. Participants gave informed consent and received course credit for their participation. This study was approved by the University of Arizona Institutional Review Board (protocol # 1811122058). As no prior studies evaluated EA and psychopathy, we were unable to anticipate expected effect sizes on which to conduct a power analysis. Therefore, our study initially aimed to recruit 200 participants as a reasonable pilot sample size. All participants without complete data were excluded before conducting analyses, leading to the final sample size stated above. However, we note that our final sample of 177 participants would afford 80% power to detect a small-to-moderate effect size (*$\rho= .21$*) relationship between EA and psychopathy, given a significance threshold of p < .05.”*

**The discussion of this limitation (p. 26) now reads as follows:**

*“Second, this study was conducted on a student sample and did not include violent or incarcerated offenders or other older and community-dwelling individuals that could include high-functioning psychopaths. Although the means for each TPM subscale in our sample were generally lower than those reported in previous forensic clinical or correctional populations [97-99], the range of scores was not dissimilar. For example, the range for our student sample’s TPM Total score was 30-111, and the range for a prisoner sample was 25-119 [98]. While the student sample might not be representative of clinical or correctional populations, it does appear to capture the continuum of psychopathic traits that may be present in the general population, as the means were similar to those reported by others with student and community groups [98-101]. Therefore, our results may not generalize to the highest levels of psychopathy, and it will be important to assess more severe populations in future studies. This also relates to limitations in our selective analyses of high-psychopathy individuals based on a median split. Namely, while those scoring above the median reflect higher levels within a student population, this may not capture high levels in a community or prison population.”*

One of the most interesting findings is that “high psychopathy (and low empathy) did not prevent individuals from displaying high EA in general.” (p. 22). I think the paper could be more strongly framed as an investigation of whether the association between psychopathy and EA is moderated by early adversity and why this is the case. Although I can see why TPM scores are treated as the outcome, it seems it might make sense to run reverse regression models with TPM predicting LEAS, including adverse experiences as a moderator. I don’t agree with the authors’ focus on the distinction between primary and secondary psychopathy since these facets were not directly assessed.

**Reply: We can understand the reviewer’s position on these issues. However, we respectfully disagree on some points. While reverse regressions could be interesting, we’re concerned about the number of analyses this would add to the paper and believe the current analyses address the question we set out to answer. We’d also prefer not to reframe the manuscript as suggested for various reasons. One important issue is that the previous round of comments from the other reviewer specifically suggested ways to better frame things in terms of primary and secondary psychopathy, and so we are hesitant to undo this framing and reverse some of the edits that led that reviewer to evaluate the manuscript more positively. The current framing is also honest with respect to certain aspects of how the study was initially conceptualized. It is our hope that this initial report will encourage others to explore the various ways emotional awareness, psychopathy, and early adversity may be inter-related.**

The authors should report the correlations between measures rather than depicting this information graphically.

**Reply: We appreciate this suggestion and recognize different preferences in reporting this information. As it stands, we do report the value of each correlation and its significance level. In our view, the correlation structure between measures is more easily digestible in the current figure than it would be within the text. Since all the statistical information that would be provided is currently in the correlation matrix, our preference is to keep things as they are presently. If the editor feels strongly, we’d be open to changing this in a subsequent revision.**

Were there any attention checks? How was the data screened/cleaned?

**Reply: We’d like to thank the reviewer for highlighting the need to further assess potential quality control issues. While attention checks were not incorporated in our report, this comment motivated us to conduct a more thorough manual examination of individual responses across all data. This identified six participants with patterns of responses on certain measures that suggested inattentive responding (i.e., identical responses across all items). Data from these participants have now been removed and all analyses were conducted again. There were no substantial changes in our results, with the exception that the interaction between LEAS and one of the early adversity measures (CECA) was now found in the 2^nd^-best model in the BF analyses (i.e., while the interaction with the other early adversity measures [CTQ] remained within the best model). The removal of these participants during quality control is now explicitly stated on p. 12:**

*“Patterns of responses on all questionnaires were manually checked to ensure a lack of random or inattentive responding. This led to the removal of partial data from six unique participants* *who showed identical responses across all items of a given scale.”*

**All LEAS responses were also thoroughly examined, as described in the methods section (p. 9).**

*“Additionally, all written responses on the LEAS were manually checked to ensure attentive completion of each item (i.e., the content of written responses directly answered the prompt provided, absence of non-identical responses across items, and lack of jumbled letters or non-words, etc.).”*

**Further, we address the inability to fully rule out inattentive responding as a limitation in the following addition (p. 27) to the discussion section:**

*“Also, some measures collected for this study relied upon self-report questionnaires and were completed without supervision. Therefore, despite efforts to ensure data quality, inattentive completion of these measures cannot be ruled out.”*

**We’d like to again thank the reviewer for their thoughtful suggestions and detailed review of our manuscript. We think the quality of the paper has been significantly improved as a result.**
